# Supplementary material for: Active DNA unwinding and transport by a membrane-adapted helicase nanopore
Source: Nat Commun. 2019 Nov 8;10:5083. doi: 10.1038/s41467-019-13047-y (PMC6841704; doi:10.1038/s41467-019-13047-y)
Supplement: Supplementary file 1 — Supplementary Information [file 41467_2019_13047_MOESM1_ESM.pdf]

Supplementary Information

Active DNA unwinding and transport by a membrane-adapted helicase nanopore

Sun et al

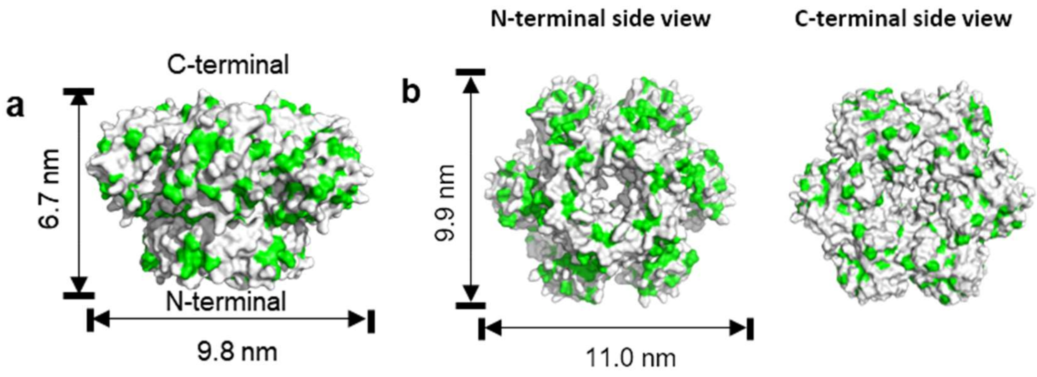

Supplementary Figure 1 The structure of truncated BPV E1. **a** Side view. **b** top view. The hydrophobic residues were coloured in green with other resides in white

Supplementary Table 1 The PCR primers sequences of two kinds of E1 proteins constructed into pGEX-6P-1 plasmid

Proteins	Primers	Sequences (5'-3')
E1 306-577	F1	CGGGATCCTTGCAGACCGAGAAAT
	R1	CCGCTCGAGTTAATGATGATGGTGATGATGCAGG TCTAAACGCCCC
E1 306-605	F2	CCGGGAGCTGCATGTGTCAGAGG
	R2	TTGCCAACCATAAAAGTCAC

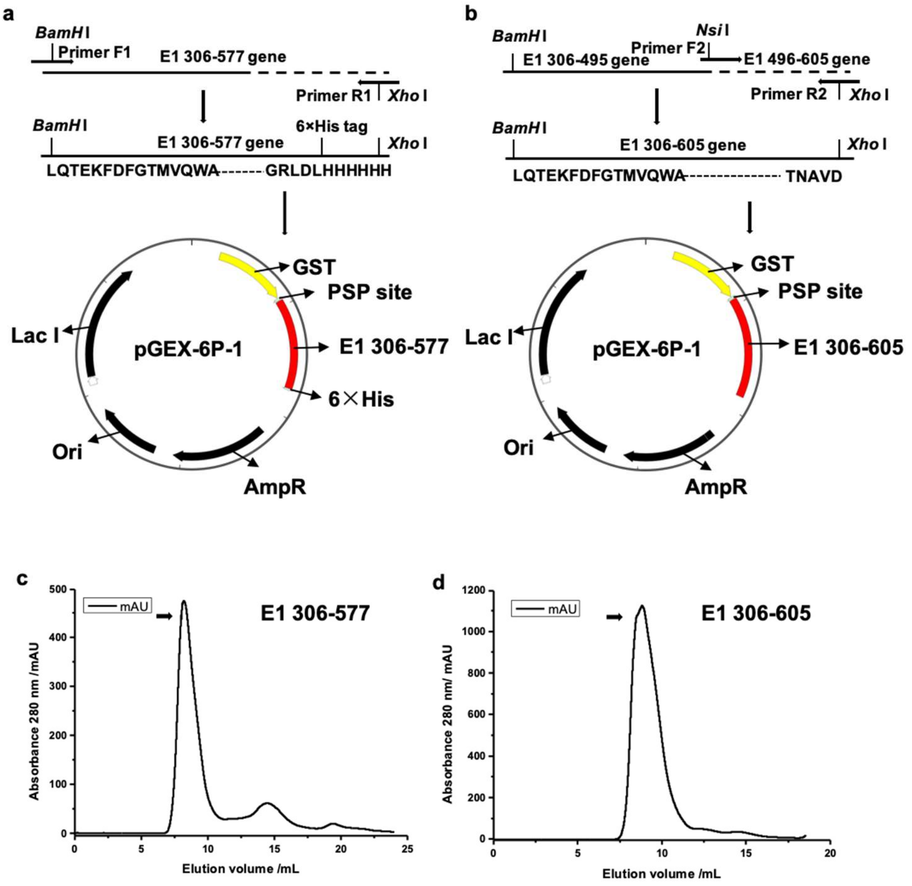

Supplementary Figure 2 Examples of two kinds of BPV E1 proteins constructed on pGEX-6P-1 plasmids and proteins purification results. **a** The gene of E1 306-577 was synthesized by PCR using primer F1 and primer R1. **b** The gene of E1 306-605 was designed according to the other enzyme cleavage site (*Nsi* I) in the existing sequence of E1 306-577 gene. **c**, **d** Elution results by gel filtration of the proteins with the elution peaks marked. Source data of c and d are provided as a Source Data file

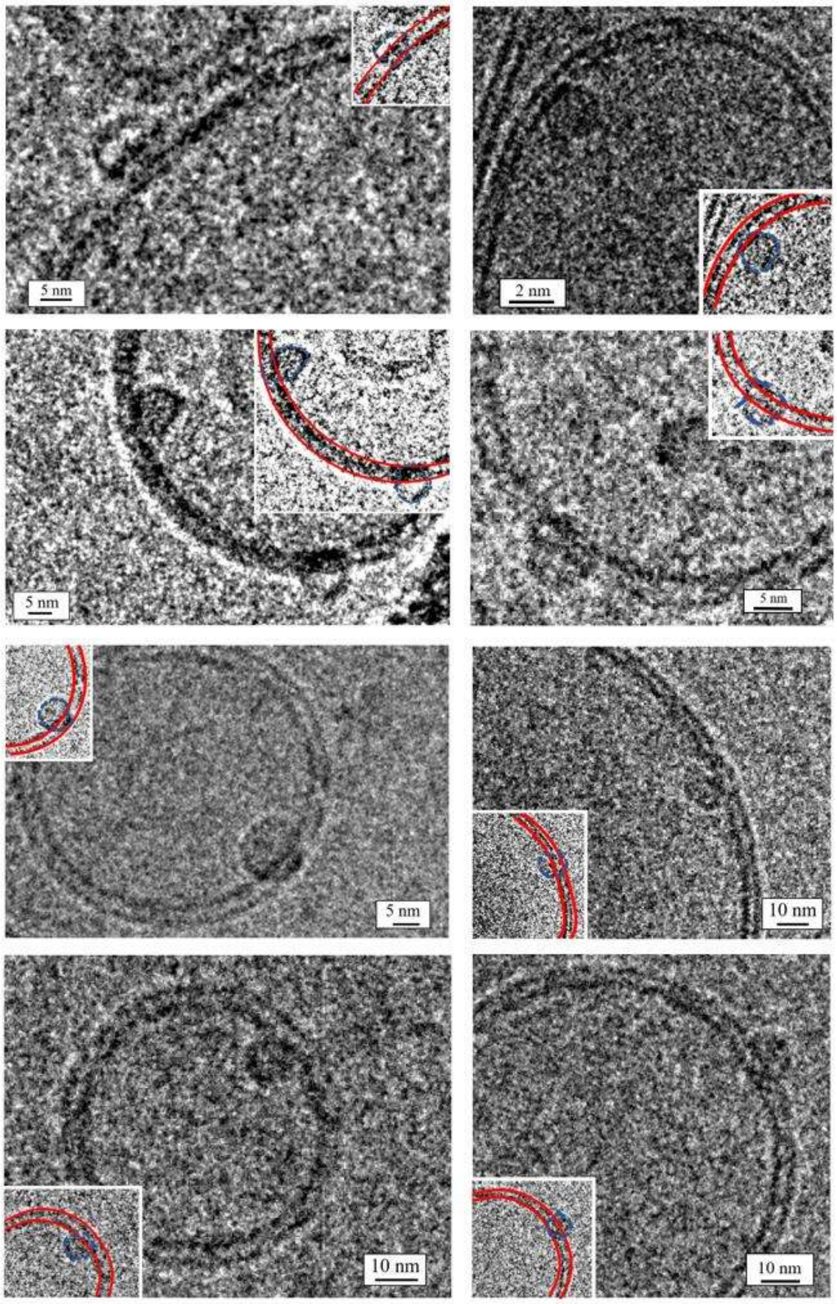

Supplementary Figure 3 The Cryo-EM images of helicase nanopore proteoliposome**.** Inset, overlaid outlines of the bilayer (red solid lines) and helicase nanopore (blue dashed lines). Source data are provided as a Source Data file

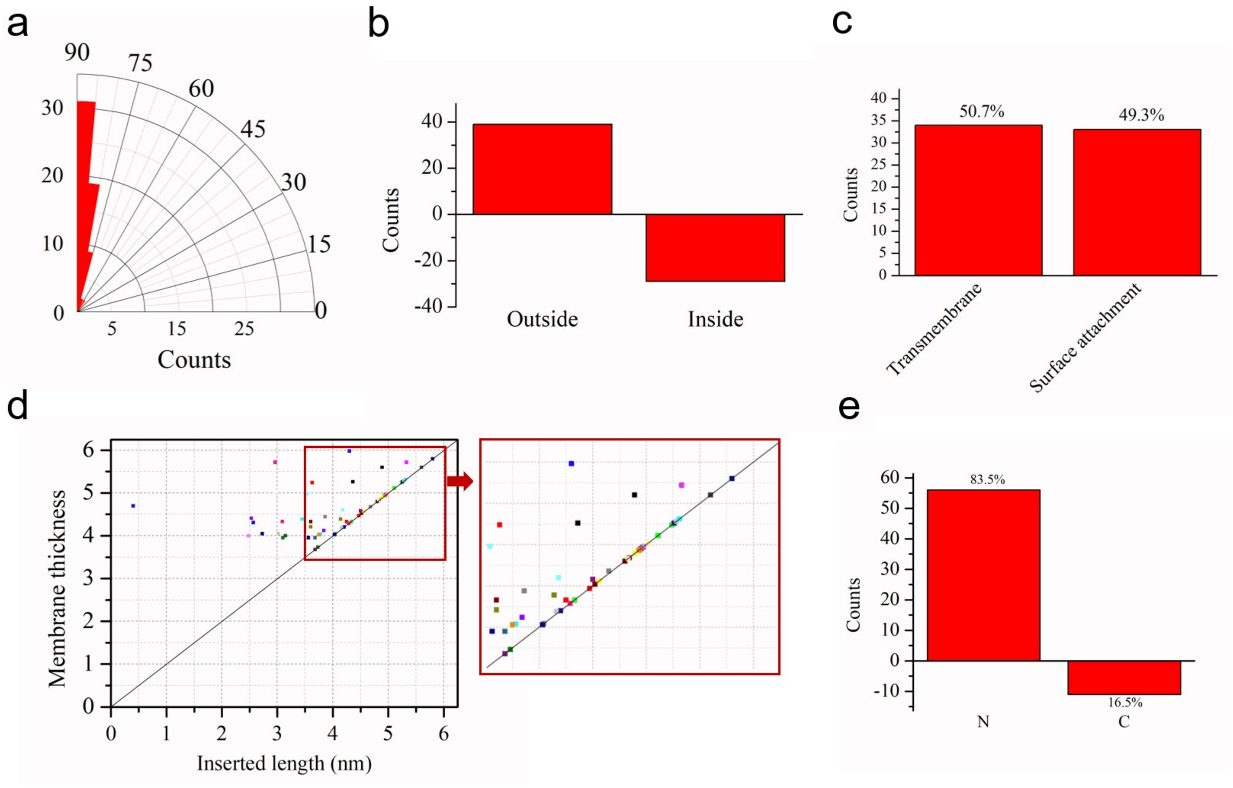

Supplementary Figure 4 The statistical results of helicase nanopore insertion into vesicles. **a** Radius plot of the histogram of helicase nanopore tilt angles measured relative to the axis normal to the bilayer plane (n=67 from 45 trials). **b** Histogram of the insertion location of inside vesicles or outside vesicles (n=67 from 45 trials). **c** Histogram of the insertion interaction of helicase nanopore with the membrane (n=67 from 45 trials). **d** Scatter plot of the thickness of lipid membrane *vs.* the inserted length, and magnified view was shown in box at right. **e** Histogram of the insertion orientation in the membrane. Source data are provided as a Source Data file

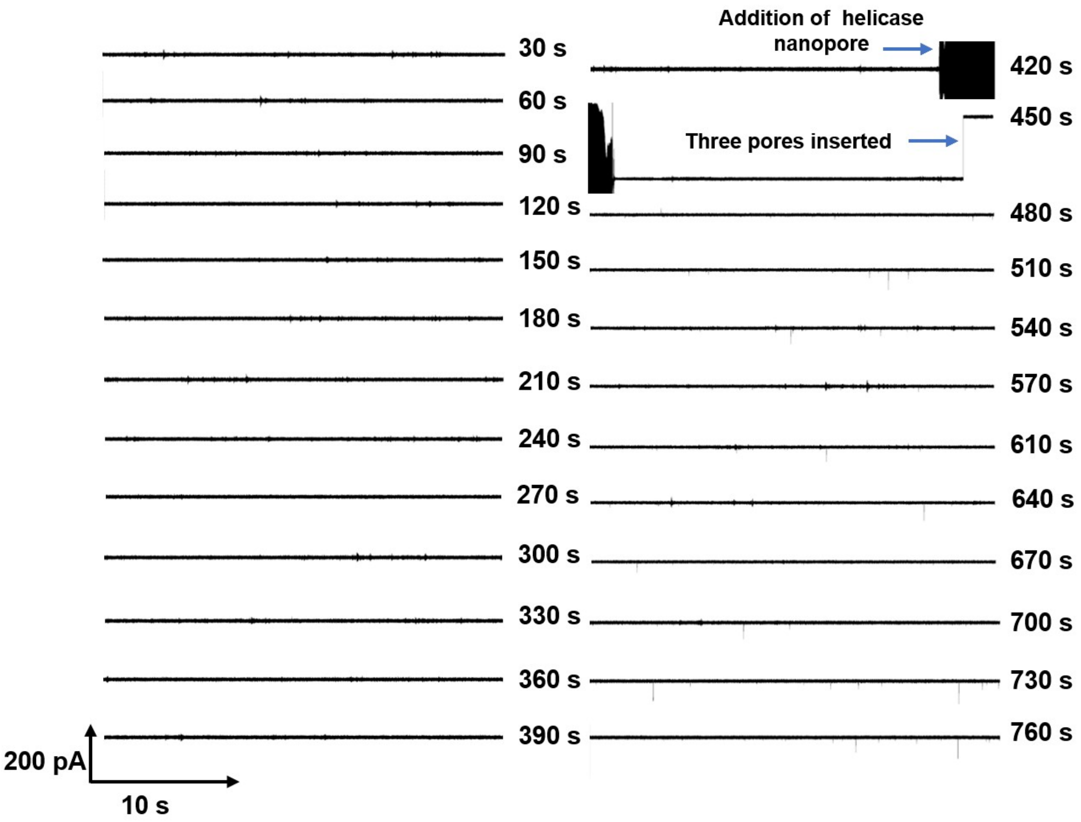

Supplementary Figure 5 Continuous current trace recording of the helicase nanopore insertion into BLM over 760 s at +70 mV. The electrolyte buffer was 5 mM HEPES, 1 M KCl, pH 7.5

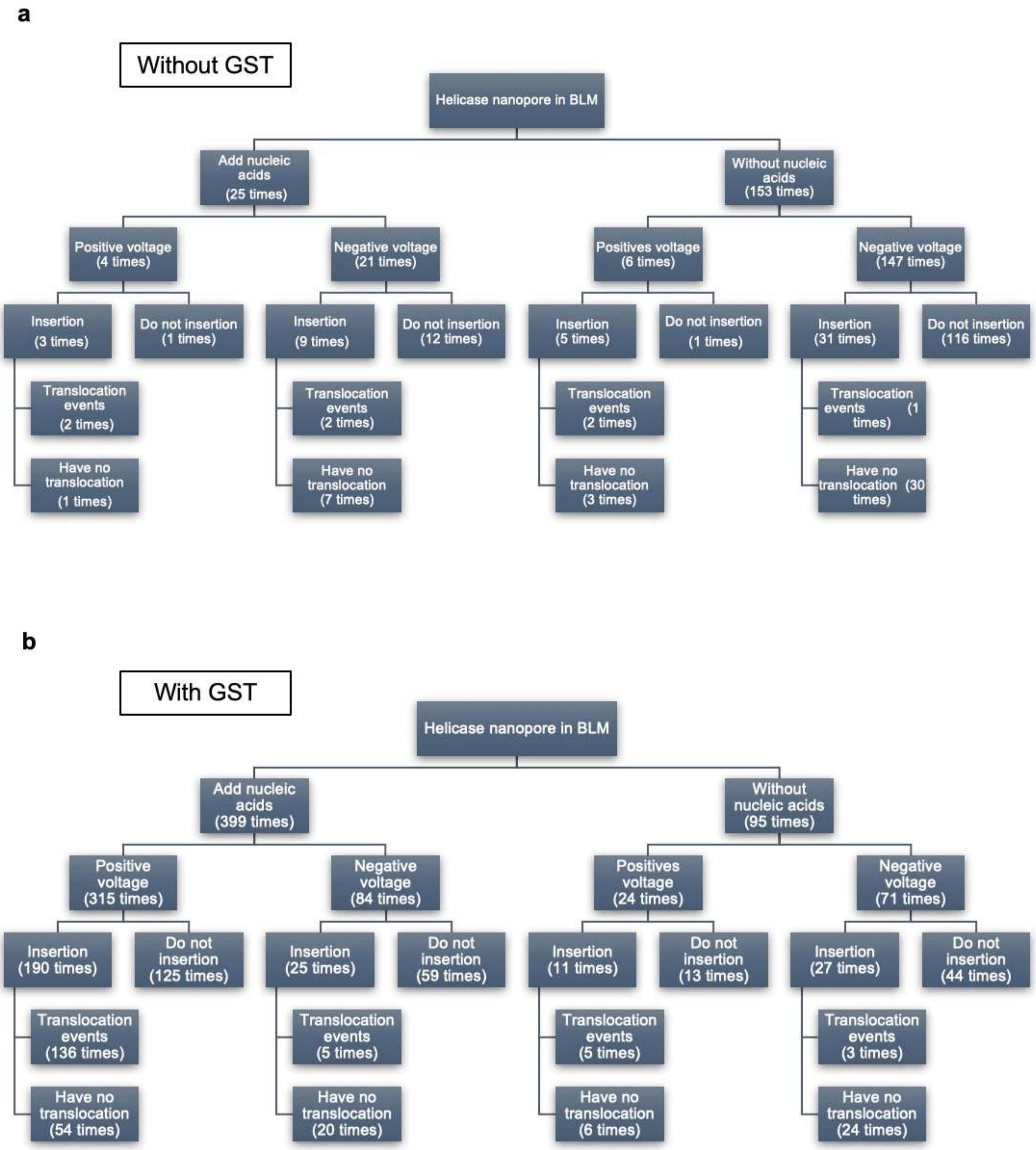

Supplementary Figure 6 Statistics of the helicase nanopores insertion into BLM and ssDNA translocation under different experiment conditions. **a** Helicase nanopore without GST-tag in high salinity buffer (1 M KCl/0.5 M KCl, 5 mM HEPES, pH 7.5) (n=178). **b** Helicase nanopore with GST-tag in high salinity buffer (1 M KCl/0.5 M KCl, 5 mM HEPES, pH 7.5) and low salinity buffer (10 mM PBS, pH 7.5) (n=672)

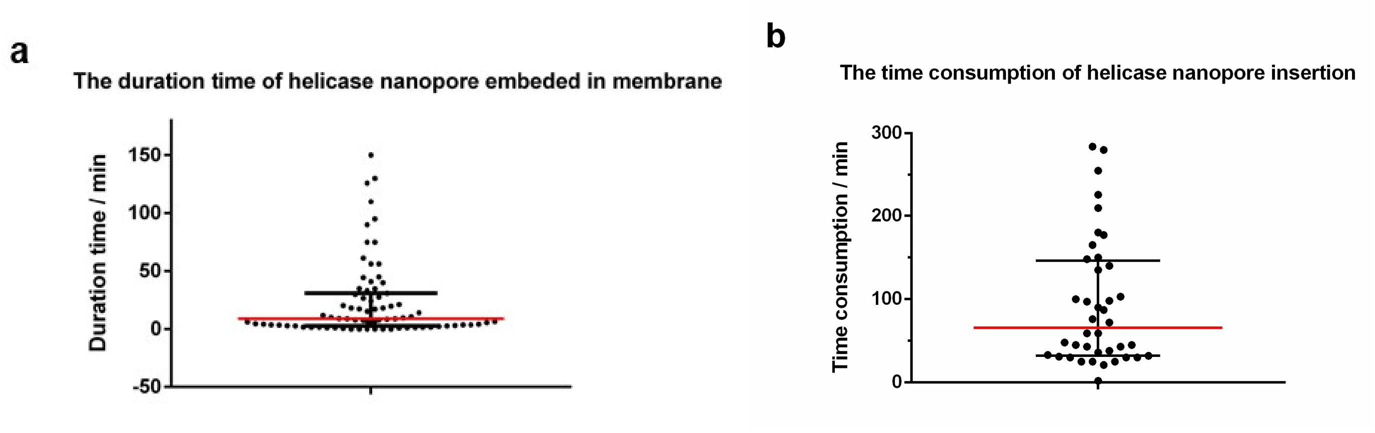

Supplementary Figure 7 The scatter diagram of the membrane incorporation time of the helicase nanopore with GST-tag. **a.** Scatter diagram of the duration time of helicase nanopore with GST-tag stable in the BLM (n=79 from 21 trials). **b** Scatter diagram of the time consumption of helicase nanopore with GST-tag to insert into the BLM. Statistical analysis was expressed as median (interquartile range). Source data are provided as a Source Data file (n=40 from 21 trials)

Supplementary Table 2 A list of the DNA used in the study

Description	Sequence	Length
Sequence A	**5'**-$(T)_9$CCCCCCTTTTTTGGGGGGTTTTTTAAAAAA$(T)_9$-**3'**	48 nt
Sequence B	**5'**-TGTTTTGCGAACTCCCCAATACTTTTCTTTTCAAATTTAAAATCTGCTCCTCACC CGCCTTTTTCTCTAATGCCTGGAAG-**3'**	80 nt
Sequence C	**5'**-/Cy5/CCTACGCCACCAGCTCCGTAGG/BHQ2/-**3'**<br>**3'**-$(T)_{20}$GGATGCGGTGGTCGAGGCATCC -**5'**	22 bp
Sequence D	**5'**-AGCTCCACCCCTCCTGGTAACCAG$(T)_{20}$-**3'**<br>**3'**-TCGAGGTGGGGAGGACCATTGGTC$(T)_{20}$-**5'**	24 bp
Sequence E	**5'**- CAGAGGACAGATAGGGCGGGTGCAAACCTTTCGCGGGGAGCAGCC$(T)_6$-**3'**<br>**3'**- GTCTCCTGTCTATCCCGCCCACGTTTGGAAAGCGCCCCTCGTCG-**5'**	44 bp
Sequence F	**5'**-CAGGCAGAGGACAGATATTTGTACTTGCATAGTCGGGTGCAAACCTTTCGCTTTG AGCAGCCATGCACAGATGAATCGGG$(T)_{20}$-**3'**<br>**3'**-GTCCGTCTCCTGTCTATAAACATGAACGTATCAGCCCACGTTTGGAAAGC GAAACTCGTCGGTACGTGTCTACTTAGCCC-5'	80 bp
Sequence G	**5'**- ACAGAATAGGGCTAACAGACAAGAGGCATA AACAGGGTAGGGTACGGGAA- **3'**<br>**3'**- ATTGTCTGTTCTCCGTATTTGTCCCATCCCATGCCCTT- **5'**	38 bp

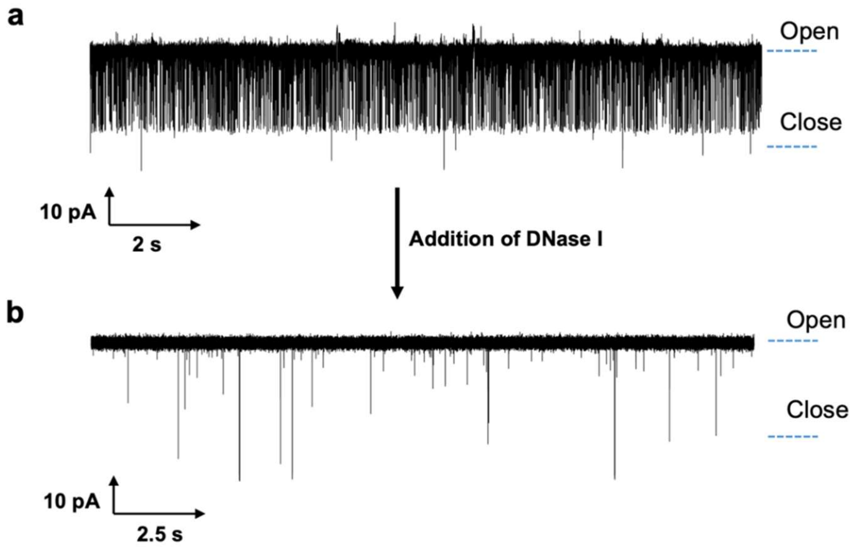

Supplementary Figure 8 The enzymatic degradation experiment of ssDNA by DNase I. **a** The typical current trace of adding ssDNA into the *cis* chamber after helicase nanopore insertion (200 nM ssDNA sequence B of 80 nt, 500 mM KCl, 5 mM HEPES, pH 7.5, under +50 mV, n=3); **b** The typical current trace of ssDNA degradation by adding 0.25 μg/mL DNase I into the *cis* chamber after 20 min (500 mM KCl, 5 mM HEPES, pH 7.5, under +50 mV, n=3). Source data are provided as a Source Data file

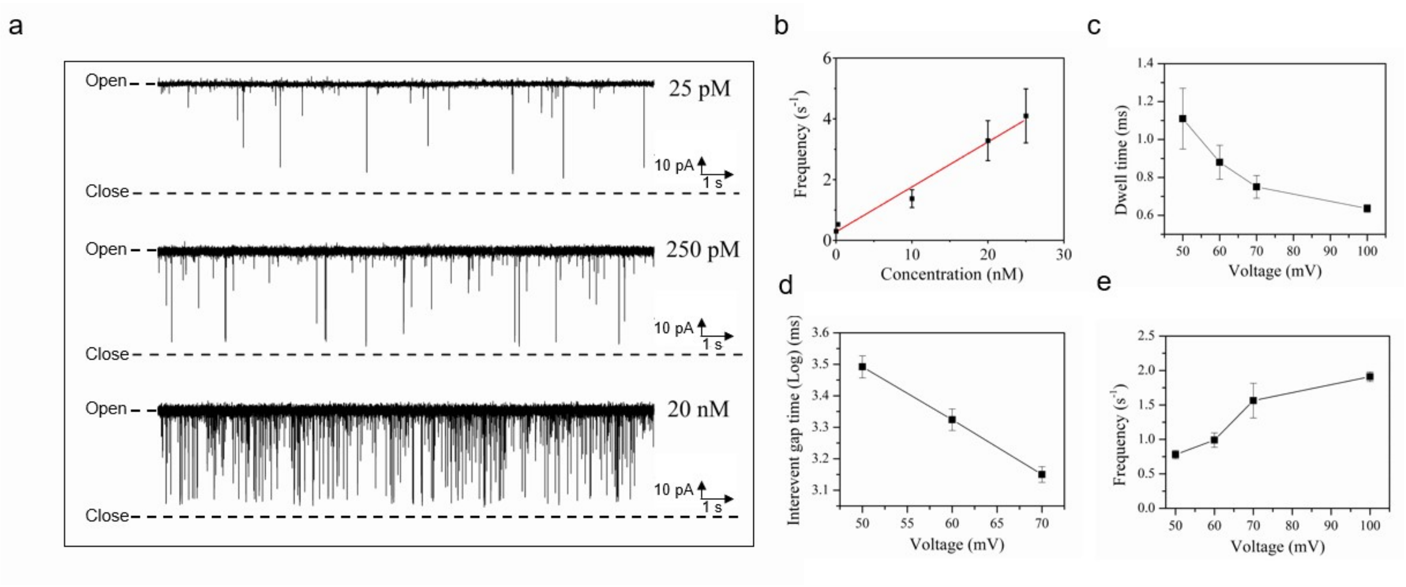

Supplementary Figure 9 The dependence of ssDNA translocation on ssDNA concentration and bias voltage. (500 mM KCl, 5 mM HEPES, pH 7.5). **a** The representative current traces of ssDNA translocation with different concentration (+100 mV). **b** The correlation between events frequency and ssDNA concentration (+50 mV, n=3). **c** The correlation between dwell time and bias voltages (250 pM ssDNA, n=3). **d** The correlation between interevent gap time of ssDNA translocation and bias voltages (250 pM ssDNA, n=3). **e** The correlation between the events frequency and applied bias voltage (250 pM ssDNA, n=3). Error bars represented the standard deviation between independent experiments. Source data are provided as a Source Data file

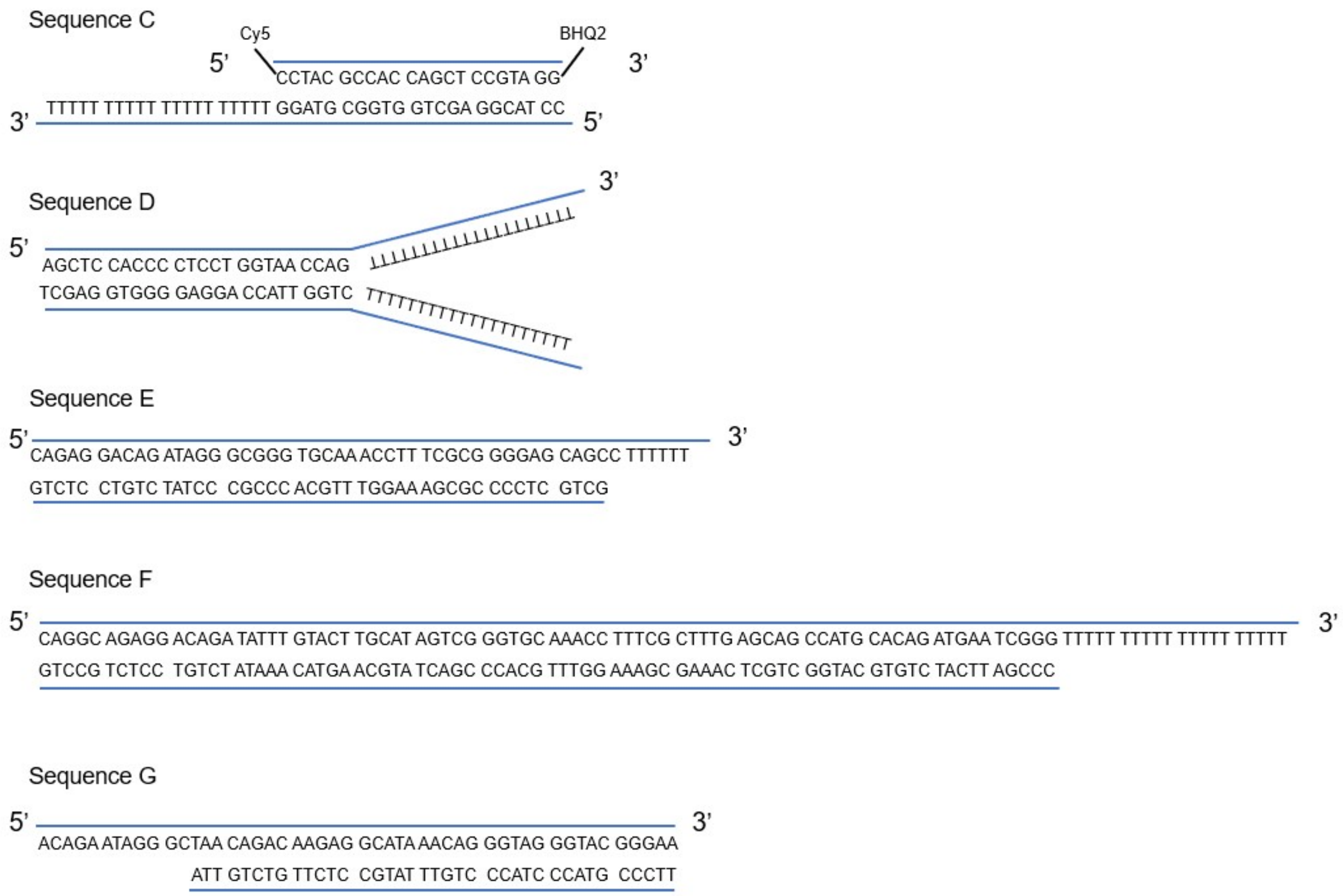

Supplementary Figure 10 The structure of different dsDNA used in the helicase activity assay (sequence C) in vitro and unwinding assay in BLM (sequence D, E, F, G)

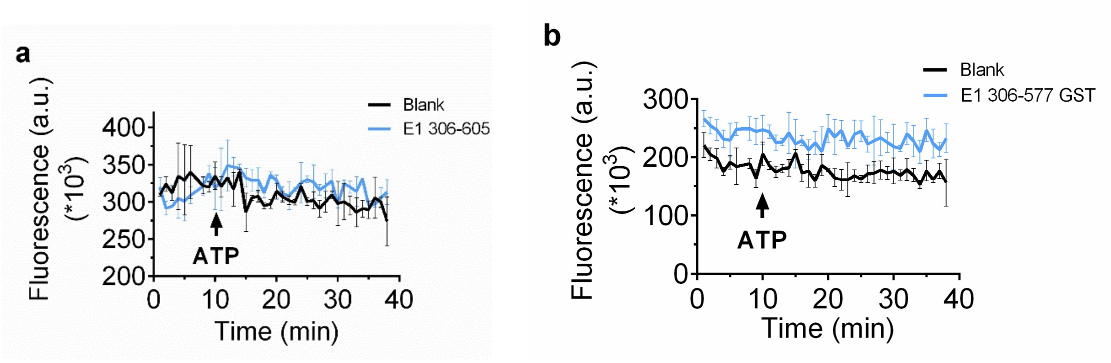

Supplementary Figure 11 Fluorescence quenching assay showing the unwinding process of dsDNA after the addition of ATP. **a** Fluorescence quenching assay of BPV E1 306-605 without GST-tag (n=3). **b** Fluorescence quenching assay of BPV E1 306-577 GST (n=3). Error bars represented the standard deviation between independent experiments. Source data are provided as a Source Data file

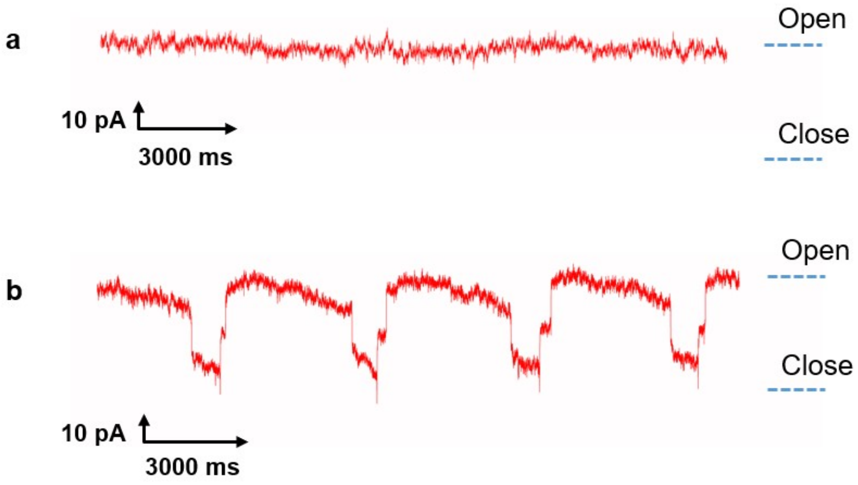

Supplementary Figure 12. The directional experiment of dsDNA translocation through the helicase nanopore. **a** No transport signals were observed at the voltage of -100 mV (dsDNA was premixed into electrolyte buffer in both *cis* and *trans* chambers). **b** dsDNA could be transported through the helicase nanopore at the voltage of +100 mV (dsDNA was premixed into electrolyte buffer in both *cis* and *trans* chambers, buffer condition: 150 mM NaCl, sequence E). Source data are provided as a Source Data file

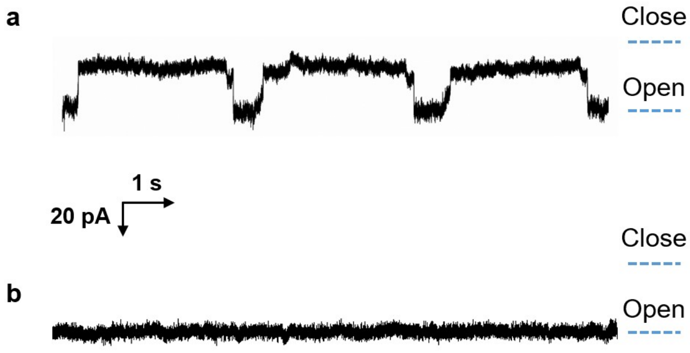

Supplementary Figure 13. The directionality of dsDNA entering helicase nanopore. **a** dsDNA translocation events could be observed after adding dsDNA with only one single-strand arm at 3' end (sequence E, buffer condition: PBS, pH 7.0, -100 mV). **b** No signals were observed when adding dsDNA with only one single-strand arm at 5' end (sequence G, buffer condition: PBS, pH 7.0, -100 mV). (n=3)

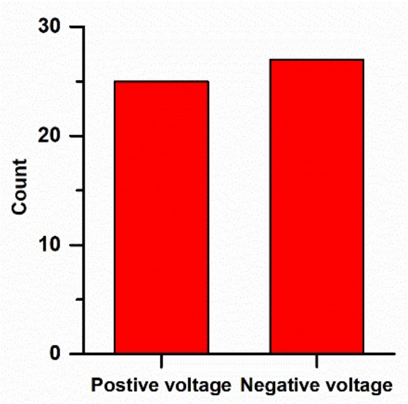

Supplementary Figure 14. The directional experiment of helicase nanopore insertion into the BLM (n=52). Source data are provided as a Source Data file

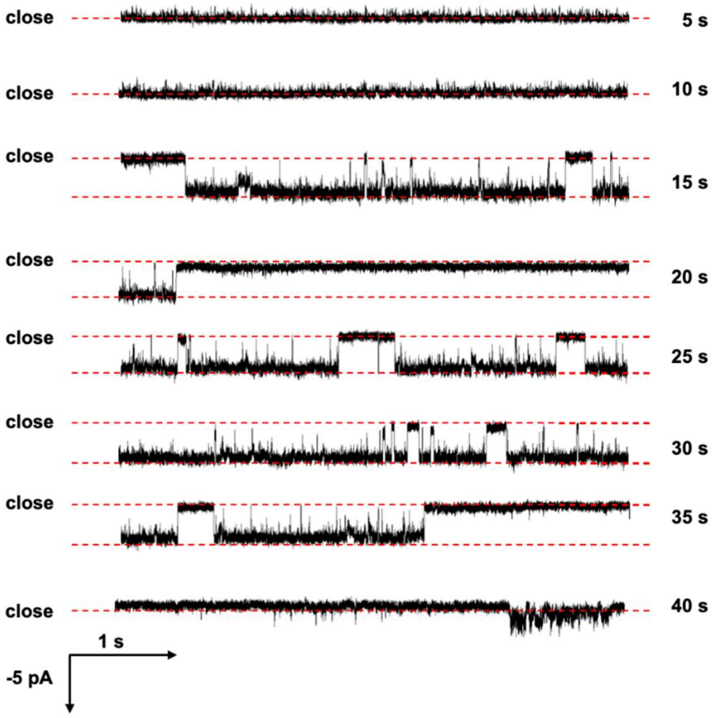

Supplementary Figure 15. Continuous current recording trace of the helicase nanopore inserted into the HEK293T cell membrane over a 40 s at a bias voltage of -60 mV at 25°C. (Extracellular solution: 145 mM NaCl, 10 mM HEPES, 10 mM glucose, 4 mM KCl, 2 mM $CaCl_2$, 1 mM $MgCl_2$, pH 7.4. Intracellular solution: 150 mM KCl, 1 mM EDTA, 10 mM HEPES, pH 7.4)

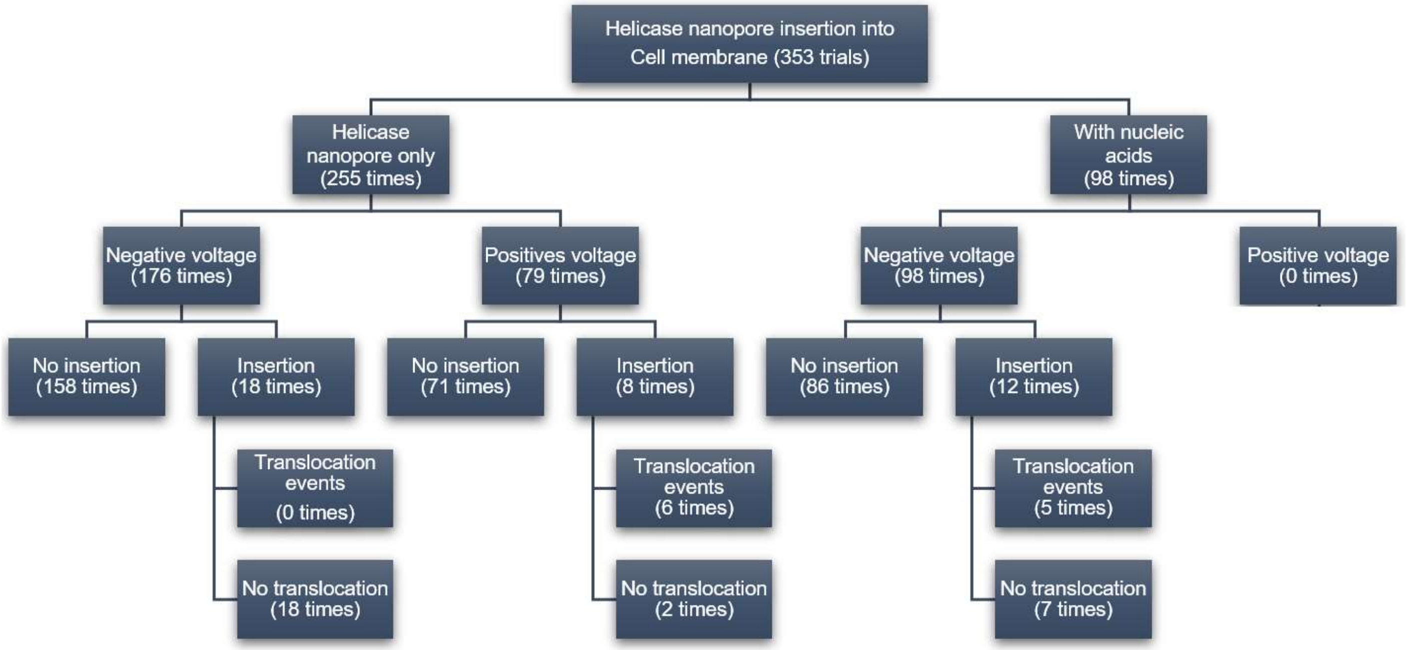

Supplementary Figure 16 Summary of 353 trials of helicase nanopore insertion in HEK293T cell membrane and translocation events under -60 and +20 mV. Extracellular solution: 145 mM NaCl, 10 mM HEPES, 10 mM glucose, 4 mM KCl, 2 mM $CaCl_2$, 1 mM $MgCl_2$, pH 7.4. Intracellular solution: 150 mM KCl, 1 mM EDTA, 10 mM HEPES, pH 7.4. 200 nM of ssDNA sequence B was used for translocation experiments

Supplementary Table 3. The mean, minimum, median and maximum of time consumption for the helicase nanopore insertion into HEK293T cells using cell patch under different conditions

**Statistic**	**Mean**	**Minimum**	**Median**	**Maximum**
**Needed time (s)**	427.25	36	298.5	1729

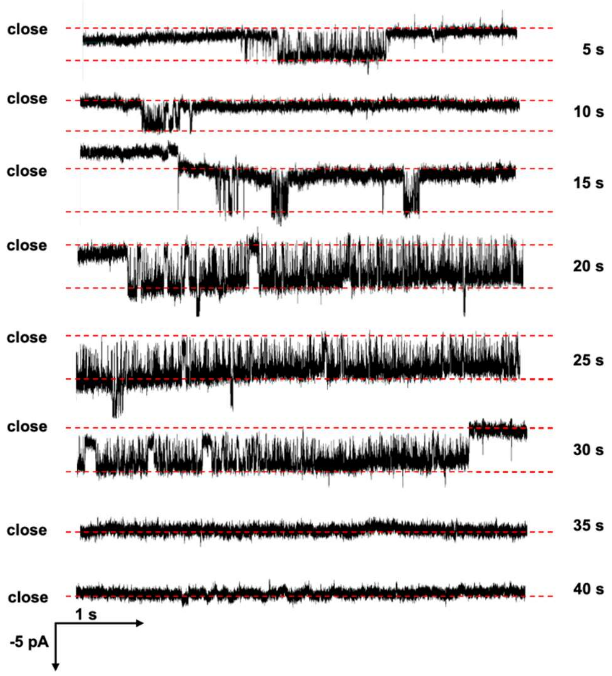

Supplementary Figure 17. Continuous current recording trace of ssDNA translocation through a helicase nanopore inserted into HEK293T cell membrane over 40 s at a voltage of -60 mV, 25°C. 200 nM ssDNA of sequence B was used. Extracellular solution: 145 mM NaCl, 10 mM HEPES, 10 mM glucose, 4 mM KCl, 2 mM $CaCl_2$, 1 mM $MgCl_2$ pH 7.4. Intracellular solution: 150 mM KCl, 1 mM EDTA, 10 mM HEPES, pH 7.4

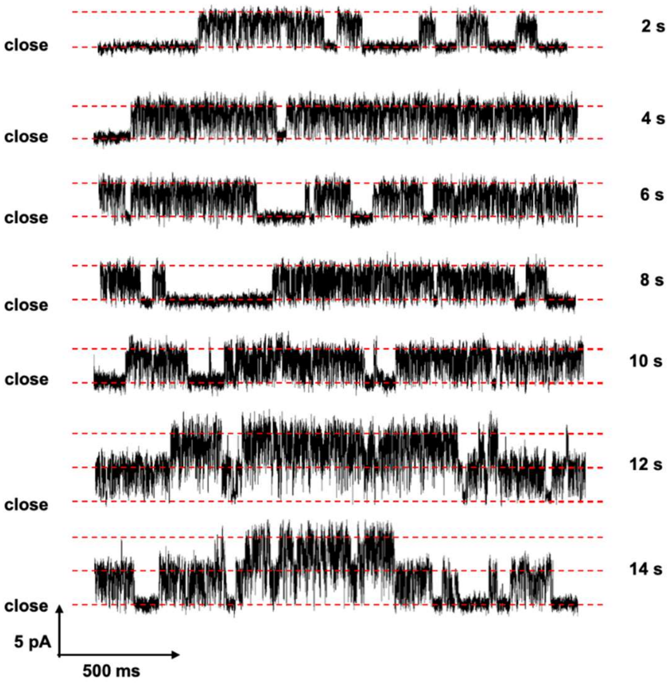

Supplementary Figure 18. Continuous current recording trace of helicase nanopore insertion into HEK293T cell membrane over 14 s at a voltage of +20 mV, 25°C. Extracellular solution: 145 mM NaCl, 10 mM HEPES, 10 mM glucose, 4 mM KCl, 2 mM $CaCl_2$, 1 mM $MgCl_2$, pH 7.4; Intracellular solution: 150 mM KCl, 1 mM EDTA, 10 mM HEPES, pH 7.4

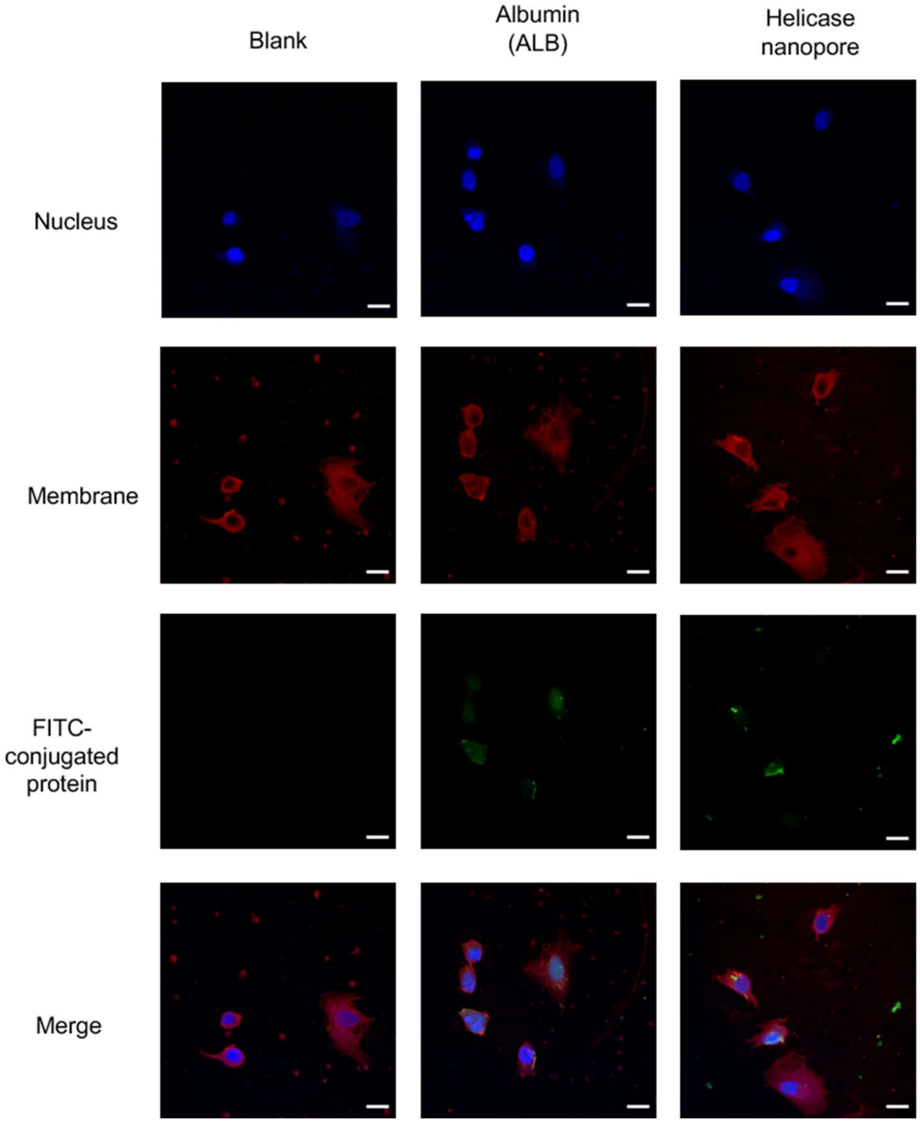

Supplementary Figure 19. Full view of confocal laser scanning microscope images showing distribution of FITC-labelled helicase nanopore in LN-229 cells. The helicase nanopore (0.2 mg/mL) was observed in cells after co-incubating with LN-229 cells for 4 h, and albumin group was used as a positive control. The results showed the helicase nanopore could incorporate with membranes and enter cells (scale bar=20 μm, lower right in the microscopy images). Source data are provided as a Source Data file

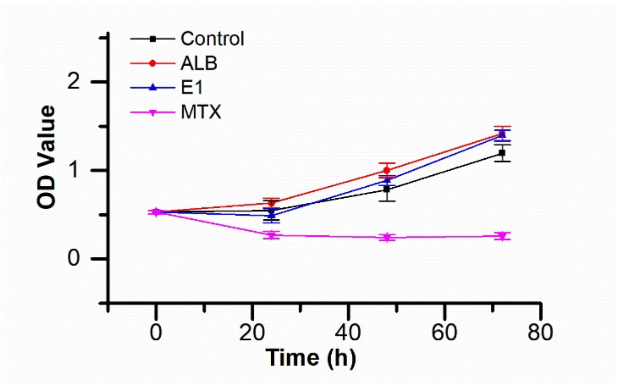

Supplementary Figure 20. Cytotoxicity assay of the helicase nanopore in LN-229 cells. Cell viability was revealed by OD values using Cell Counting Kit-8 (CCK-8) method. During the observation time (0 h-72 h), helicase nanopore (0.5 mg/mL) didn't show cytotoxicity in LN-229 cells, when compared with positive control (Methotrexate, MTX). The growth curve of helicase nanopore almost overlapped with the control group and negative control group (Albumin, ALB). Error bars represented the standard deviation between independent experiments. Source data are provided as a Source Data file
