# Supplementary material for: Active DNA unwinding and transport by a membrane-adapted helicase nanopore
Source: Nat Commun. 2019 Nov 8;10:5083. doi: 10.1038/s41467-019-13047-y (PMC6841704; doi:10.1038/s41467-019-13047-y)
Supplement: Supplementary file 3 — Reporting Summary [file 41467_2019_13047_MOESM3_ESM.pdf]

nature research

Corresponding author(s): Jia GENG, Guangwen LU, Lei ZHANG

Last updated by author(s): Oct 13, 2019

Reporting Summary

Nature Research wishes to improve the reproducibility of the work that we publish. This form provides structure for consistency and transparency in reporting. For further information on Nature Research policies, see Authors & Referees and the Editorial Policy Checklist .

Statistics

For all statistical analyses, confirm that the following items are present in the figure legend, table legend, main text, or Methods section.

n/a	Confirmed	
☐	☒	The exact sample size (n) for each experimental group/condition, given as a discrete number and unit of measurement
☐	☒	A statement on whether measurements were taken from distinct samples or whether the same sample was measured repeatedly
☒	☐	The statistical test(s) used AND whether they are one- or two-sided<br>*Only common tests should be described solely by name; describe more complex techniques in the Methods section.*
☒	☐	A description of all covariates tested
☒	☐	A description of any assumptions or corrections, such as tests of normality and adjustment for multiple comparisons
☐	☒	A full description of the statistical parameters including central tendency (e.g. means) or other basic estimates (e.g. regression coefficient) AND variation (e.g. standard deviation) or associated estimates of uncertainty (e.g. confidence intervals)
☒	☐	For null hypothesis testing, the test statistic (e.g. F, t, r) with confidence intervals, effect sizes, degrees of freedom and P value noted<br>*Give P values as exact values whenever suitable.*
☒	☐	For Bayesian analysis, information on the choice of priors and Markov chain Monte Carlo settings
☒	☐	For hierarchical and complex designs, identification of the appropriate level for tests and full reporting of outcomes
☒	☐	Estimates of effect sizes (e.g. Cohen's d, Pearson's r), indicating how they were calculated

Our web collection on statistics for biologists contains articles on many of the points above.

Software and code

Policy information about availability of computer code

Data collection	ZEN 2.3 lite for Confocal microscopy imaging;<br>Bio-Rad CFX Manager 3.1 for Q-pcr detection;<br>Ascent Software version 2.6 for CCK-8 kit detection;<br>Clampex version10.6.0.13 for Axon patch clamp data collection;<br>PatchMaster v2x73.5 for HEKA patch clamp data collection;<br>Image Micro-Manager 1.4.23 for cell imaging;<br>Image lab 5.2.1 for gel imaging;<br>High-sensitivity 4Kx4K pixel FEI CETA CMOS camera for Cryo-EM micrographs;
Data analysis	OriginPro 8.5.1 SR2, and Clampfit version 10.6.1.1 for single channel recording data analysis;<br>OriginPro 9.0.0 (32-bit) b45 for the analysis of elution results by gel filtration of proteins;<br>GraphPad Prism 6.01 for scattering plot;<br>ImageJ 1.52n for measurement of proteoliposome vesicle size.

For manuscripts utilizing custom algorithms or software that are central to the research but not yet described in published literature, software must be made available to editors/reviewers. We strongly encourage code deposition in a community repository (e.g. GitHub). See the Nature Research guidelines for submitting code & software for further information.

Data

Policy information about availability of data

All manuscripts must include a data availability statement. This statement should provide the following information, where applicable:

- Accession codes, unique identifiers, or web links for publicly available datasets
- A list of figures that have associated raw data
- A description of any restrictions on data availability

The source data underlying Figs. 1c, 1e, 2b-i, 3a-l, 4a-h and Supplementary Figs2c-d, 3, 4, 7, 8, 9, 11, 12, 13, 14, 19, 20 are provided as a Source Data file (https://figshare.com/s/c87ceb4d356076d0d3a7)

Field-specific reporting

Please select the one below that is the best fit for your research. If you are not sure, read the appropriate sections before making your selection.

[x] Life sciences [ ] Behavioural & social sciences [ ] Ecological, evolutionary & environmental sciences

For a reference copy of the document with all sections, see nature.com/documents/nr-reporting-summary-flat.pdf

Life sciences study design

All studies must disclose on these points even when the disclosure is negative.

Sample size	Each data point contains at least three different repeats, and the detailed sample size in each figures was described in the manuscript.
Data exclusions	No data from the experiments was excluded.
Replication	All the experiments in the manuscript were repeated at least for 3 times, and the number of experiments were described.
Randomization	Not applicable.
Blinding	Investigators were not blinded.

Reporting for specific materials, systems and methods

We require information from authors about some types of materials, experimental systems and methods used in many studies. Here, indicate whether each material, system or method listed is relevant to your study. If you are not sure if a list item applies to your research, read the appropriate section before selecting a response.

Materials & experimental systems

n/a	Involved in the study	
[x]	[ ]	Antibodies
[ ]	[x]	Eukaryotic cell lines
[x]	[ ]	Palaeontology
[x]	[ ]	Animals and other organisms
[x]	[ ]	Human research participants
[x]	[ ]	Clinical data

Methods

n/a	Involved in the study	
[x]	[ ]	ChIP-seq
[x]	[ ]	Flow cytometry
[x]	[ ]	MRI-based neuroimaging

Eukaryotic cell lines

Policy information about cell lines

Cell line source(s)	LN-229 cell line was purchased from ATCC; HEK293T cell line was purchased from Cell Bank of the Typical Culture Preservation Committee, Chinese Academy of Sciences (SGST.CN).
Authentication	LN-229 cell lines was authenticated through STR identification; HEK293T cell lines was not performed any identification by ourselves, but Chinese Academy of Sciences stated that all cell lines they sold was authenticated through STR identification.
Mycoplasma contamination	We confirm that all cell lines tested negative for Mycoplasma contamination.
Commonly misidentified lines (See ICLAC register)	*Name any commonly misidentified cell lines used in the study and provide a rationale for their use.*
